# Association between Advanced Lung Cancer Inflammation Index and 30-day mortality in patients with spontaneous intracerebral hemorrhage: a retrospective cohort study

**DOI:** 10.3389/fneur.2025.1612333

**Published:** 2025-07-28

**Authors:** Jianzhong Ding, Jiquan Zhang, Wanqiu Lv, Yangchun Xiao, Fang Fang, Wenchao Ma, Yu Zhang, Xiaoli Zhong

**Affiliations:** ^1^School of Basic Medical Sciences and School of Nursing, Chengdu University, Chengdu, Sichuan, China; ^2^Department of Nursing, Deyang People’s Hospital, Deyang, Sichuan, China; ^3^Department of Neurosurgery, Clinical Medical College and Affiliated Hospital of Chengdu University, Chengdu University, Chengdu, Sichuan, China; ^4^Department of Neurosurgery, West China Hospital, Sichuan University, Chengdu, Sichuan, China; ^5^Department of Neurosurgery, Deyang People’s Hospital, Deyang, Sichuan, China

**Keywords:** spontaneous intracerebral hemorrhage, Advanced Lung Cancer Inflammation Index, 30-day mortality, neurocritical care, composite biomarker

## Abstract

**Background and purpose:**

Spontaneous intracerebral hemorrhage (ICH) is a leading cause of stroke-related mortality, with early outcomes significantly influenced by inflammation and malnutrition. However, the prognostic value of composite biomarkers, which integrate multiple factors such as inflammation and nutritional status, remains insufficiently explored, particularly in the context of early outcomes following ICH. This study explores the association between the Advanced Lung Cancer Inflammation Index (ALI) and 30-day mortality in ICH patients, evaluating its prognostic value as a composite biomarker that integrates body mass index (BMI), serum albumin, and neutrophil-to-lymphocyte ratio (NLR).

**Methods:**

A retrospective cohort study was conducted on 2,459 ICH patients from a single institution, West China Hospital, between January 2009 and June 2019. ALI was calculated using the formula ALI = (BMI × Alb)/NLR, and patients were stratified into quartiles based on ALI scores. The primary outcome was 30-day mortality, and logistic regression analysis was employed to evaluate the association between ALI and mortality.

**Results:**

The 30-day mortality rate was 23.3% (*n* = 574) in the cohort, with higher ALI scores significantly correlating with lower mortality. Patients in the highest ALI quartile (Q4) had 55% lower odds of mortality compared to those in the lowest quartile (Q1) (adjusted odds ratio [*AOR*]: 0.45, 95% *CI*: 0.31–0.63, *p* < 0.001). Each 1-standard deviation increase in ALI (as a continuous variable) was associated with a 5% reduction in mortality odds (*AOR*: 0.95, 95% *CI*: 0.93–0.96, *p* < 0.001). ROC curve analysis demonstrated that ALI outperformed individual biomarkers (BMI, albumin, and NLR) in predicting mortality, with an AUC of 0.681, which further improved to 0.811 when ALI was integrated into the ICH prediction model.

**Conclusion:**

ALI is a reliable composite biomarker, with higher ALI scores being significantly associated with reduced 30-day mortality in patients with ICH.

## Introduction

Spontaneous ICH is a severe neurological condition with high early mortality rates. Accounting for approximately 10–20% of all stroke cases globally, it presents a formidable clinical challenge (1). Despite advances in stroke care, the prognosis of ICH remains poor, with 30-day mortality rates ranging between 30 and 40% (2). This significant high early mortality reflects a combination of primary brain processes, including hematoma expansion and mass effect, and secondary injury mechanisms such as neuroinflammation, cerebral edema, and systemic metabolic disturbances (3). Early risk stratification is critical for guiding clinical decisions and improving patient outcomes, yet reliable prognostic biomarkers remain lacking (4).

Inflammation and malnutrition have emerged as pivotal determinants of early mortality (5). Neuroinflammation exacerbates blood–brain barrier disruption, amplifies cerebral edema, and accelerates secondary neuronal injury, while systemic inflammatory responses can further worsen neurological damage (6). Malnutrition, which compromises immune competence and tissue repair, has also been linked to poor outcomes (7). While biomarkers such as BMI, serum albumin, and the NLR have been individually associated with ICH outcomes, their isolated use often provides limited predictive utility (8). This limitation highlights the need for composite biomarkers that integrate nutritional and inflammatory status for enhanced prognostic accuracy (9).

The ALI, a composite biomarker combining BMI, serum albumin, and NLR, was initially developed as a prognostic tool in advanced lung cancer (10). ALI reflects both nutritional reserves and systemic inflammatory burden, offering a holistic measure of systemic health. Recent studies have highlighted its prognostic value in conditions such as sepsis and cardiovascular diseases (11, 12). In the context of ICH, where malnutrition and inflammation are synergistic drivers of secondary brain injury, ALI may hold potential as a novel biomarker for early mortality risk stratification (13).

Despite its theoretical advantages, the role of ALI in predicting short-term outcomes in ICH remains insufficiently explored. Furthermore, whether ALI offers incremental predictive value over its individual components has not been systematically investigated. To address these gaps, this retrospective cohort study evaluates the association between ALI and 30-day mortality in patients with ICH. Additionally, the study compares ALI’s prognostic performance to its individual components, with the goal of determining its clinical utility in neurocritical care.

## Methods

### Study design and population

This retrospective cohort study was conducted at West China Hospital, Sichuan University in China, focusing on patients admitted with ICH between January 2009 and June 2019. A total of 6,254 patients were initially screened. The study was conducted in compliance with the Declaration of Helsinki and received approval from the ethics committee of West China Hospital prior to initiation. Informed consent from participants was waived. The inclusion criteria were as follows: (1) age ≥18 years, (2) radiologically confirmed ICH via computed tomography (CT) or magnetic resonance imaging (MRI), and (3) availability of complete clinical and laboratory data within 24 h of admission.

The exclusion criteria were applied systematically, as detailed in the Supplementary Figure 1. Initially, patients with traumatic brain injury (*n* = 2,185) were excluded, followed by those lacking essential clinical data, including NLR (*n* = 1,514), serum albumin levels (*n* = 75), or BMI (*n* = 21). After applying these criteria, a total of 2,459 patients were included in the final analysis.

### Data collection

Baseline characteristics were collected from electronic medical records, including demographic data, comorbidities, and clinical parameters. Laboratory data, including serum albumin, neutrophil count, and BMI, were collected within 24 h of admission.

The primary outcome was 30-day mortality, defined as death occurring within 30 days after the onset of ICH. Mortality data were obtained through hospital records or follow-up phone interviews conducted by trained staff.

### Definition and calculation of ALI

The ALI was calculated using the formula:


$$\mathrm{ALI}=\frac{\mathrm{BMI}\times \mathrm{Alb}}{\mathrm{NLR}}$$

In this formula, BMI refers to body mass index (kg/m^2^), calculated as weight (kg) divided by the square of height (m^2^); Alb indicates serum albumin concentration (g/dL); and NLR represents the neutrophil-to-lymphocyte ratio, calculated as the absolute neutrophil count divided by the absolute lymphocyte count.

Patients were stratified into quartiles (Q1–Q4) based on the distribution of ALI scores, with Q1 representing the lowest ALI scores and Q4 the highest. This stratification allowed for the evaluation of the relationship between ALI and 30-day mortality.

### Outcomes

The primary outcome of this study was 30-day mortality, defined as death occurring within 30 days from the onset of ICH. Mortality data were obtained from hospital records and verified through follow-up phone interviews conducted by trained staff to ensure accuracy.

### Statistical analysis

All statistical analyses were conducted using R software (version4.4.2), with a two-tailed *p*-value < 0.05 considered statistically significant. Continuous variables were summarized as mean ± standard deviation (SD) or median (interquartile range, IQR) based on data distribution, and categorical variables were presented as frequencies and percentages. As ALI values were not normally distributed (Shapiro–Wilk test, *p* < 0.001), and no standardized clinical threshold has been established for ICH, patients were stratified into quartiles (Q1–Q4) based on the distribution of ALI within the study cohort. This data-driven approach, consistent with prior studies in oncologic and stroke populations, allowed for an exploratory evaluation of risk gradients across the spectrum of inflammation and nutritional status (10, 14). Baseline characteristics were compared across ALI quartiles using one-way analysis of variance (ANOVA) or Kruskal–Wallis tests for continuous variables and chi-square tests for categorical variables. Logistic regression models were used to assess the association between ALI and 30-day mortality, adjusting for key covariates (15). Both univariate and multivariable models were constructed to estimate odds ratios (*OR*s) and their corresponding 95% confidence intervals (*CI*s) (8).

For univariate analysis, *OR*s were calculated for ALI scores (as a continuous variable), ALI quartiles, and other potential predictors, including demographic, clinical, and laboratory variables. For the multivariable logistic regression model, a manual variable selection strategy was employed. This approach reflects the complexity of ICH outcomes, where variables must be selected not only based on statistical significance but also on clinical relevance and prior evidence (4, 16–18). Guided by the existing literature, nine key covariates were included in the final model: age, smoking, alcohol, hypertension, diabetes, GCS score, hematoma size, hematoma infratentorial, and hematoma intraventricular. The final model quantified the independent association between ALI and 30-day mortality.

Kaplan–Meier survival analysis was conducted to compare 30-day survival probabilities across ALI quartiles. The log-rank test was used to assess differences in survival curves, and risk tables were included to display the number of patients at risk over time. Restricted cubic splines (RCS) were used to explore potential nonlinear relationships between ALI (as a continuous variable) and 30-day mortality. The spline models were adjusted for key covariates, and both the overall significance and the nonlinearity significance were reported. Results were visualized as odds ratios across the range of ALI scores.

To evaluate the predictive performance of ALI, two primary analyses were conducted using receiver operating characteristic (ROC) curves. First, the standalone accuracy of ALI in predicting 30-day mortality was compared with that of its individual components, including BMI, albumin, NLR, lymphocyte count, and neutrophil count (19). Second, a baseline prognostic model was constructed using multivariable logistic regression, incorporating five established predictors from the conventional ICH score: age, GCS score, hematoma size, intraventricular hemorrhage, and infratentorial location (4). Extended models were subsequently developed by adding ALI or other inflammation-based indices such as neutrophil-to-albumin ratio (NAR), platelet-to-lymphocyte ratio (PLR), and lymphocyte-to-monocyte ratio (LMR), selected for their established relevance in inflammation and clinical prognosis (20). The area under the curve (AUC) was calculated for each model, and pairwise comparisons were performed using the DeLong test to assess improvements in discrimination. In addition, a category-based Net Reclassification Improvement (NRI) analysis was conducted to quantify the extent to which ALI enhanced risk classification for 30-day mortality beyond AUC measures (21).

To evaluate potential selection bias introduced by missing data, baseline characteristics were compared between patients included in the final analysis (*n* = 2,459) and those excluded due to incomplete records (*n* = 3,795). Variables assessed included age, GCS score, hypertension, diabetes, hematoma size, and hematoma location. In addition, to examine the robustness of the observed association between ALI and 30-day mortality, a sensitivity analysis was performed using the E-value, which estimates the minimum strength of association that an unmeasured confounder would need to explain away the observed effect (22). The E-value was calculated using a validated online tool^1^ (23), which has been widely applied in observational research.

Subgroup analyses were conducted to determine whether the association between ALI and 30-day mortality was consistent across clinically relevant subgroups. To facilitate analysis, the ALI was divided into two groups, ALI_Low (≤ median) and ALI_High (> median), based on its median value within the study cohort. Age was categorized into two groups: older adults (≥65 years) and younger adults (<65 years). The GCS score was dichotomized into two groups: ≤8 and >8. Hematoma size was categorized into two groups: ≤16 mL and >16 mL. Hematoma location was classified as supratentorial or infratentorial. Additional subgroups included sex, smoking status, alcohol use, diabetes, and hypertension. Interaction terms were included in the logistic regression models to test for effect modification across these subgroups, providing insights into whether the prognostic value of ALI varied across different clinical and demographic characteristics.

## Results

Among the 6,254 patients with ICH initially screened, 2,459 patients met the inclusion criteria and were included in the final analysis (Table 1). Patients were stratified into quartiles based on ALI scores, with Q1 representing the lowest ALI scores and Q4 the highest. Significant differences were observed across the ALI quartiles in key demographic, clinical, and laboratory characteristics. Patients in Q4 had higher BMI (25.3 ± 4.7 vs. 22.4 ± 3. 8, *p* < 0.001) and albumin levels (4.0 ± 0.5 vs. 3.6 ± 0.7 g/dL, *p* < 0.001), but lower neutrophil-to-lymphocyte ratios (5.1 ± 1.1 vs. 15.7 ± 6.8, *p* < 0.001) compared to Q1. Neurologically, Q4 patients exhibited higher GCS scores (12.4 ± 3.6 vs. 8.5 ± 3.9, *p* < 0.001) and smaller hematoma sizes (15.4 ± 19.3 mL vs. 31.8 ± 28.1 mL, *p* < 0.001). Furthermore, the proportion of patients with intraventricular hematomas differed significantly across quartiles (31.1% in Q1 vs. 17.6% in Q4, *p* < 0.001), indicating a lower prevalence of severe baseline injury in Q4. No significant differences were observed in sex distribution (*p* = 0.447), or diabetes prevalence (*p* = 0.785) across ALI quartiles. These findings indicate that patients in the highest ALI quartile generally presented with better nutritional status, lower systemic inflammation, and less severe neurological injury.

**Table 1:** Baseline characteristics by ALI quartiles.

Characteristics	Advanced Lung Cancer Inflammation Index (ALI)				*p*-value
	Q1 (*N* = 615)	Q2 (*N* = 615)	Q3 (*N* = 614)	Q4 (*N* = 615)	
Demographics					
Age, years (mean ± SD)	58.6 ± 15.1	56.5 ± 14.0	56.3 ± 14.2	54.4 ± 14.2	<0.001
Sex, male % (*N*)	66.3 (408)	67.8 (417)	67.8 (416)	70.6 (434)	0.447
Smoking % (*N*)	66.5 (411)	67.6 (417)	67.6 (417)	70.5 (435)	0.022
Alcohol % (*N*)	27.6 (170)	32.8 (202)	30.0 (184)	35.8 (220)	0.014
BMI, kg/m^2^ (mean ± SD)	22.4 ± 3.8	24.2 ± 3.4	24.8 ± 4.3	25.3 ± 4.7	<0.001
SBP (mean ± SD)	165.1 ± 34.0	164.0 ± 33.4	161.7 ± 31.6	154.3 ± 30.4	<0.001
Comorbidities % (*N*)					
Diabetes	9.4 (58)	11.2 (69)	10.3 (63)	10.4 (64)	0.785
Hypertension	76.6 (471)	74.6 (459)	74.3 (456)	67.5 (415)	0.002
Hematoma characteristics					
Hematoma intraventricular % (*N*)	31.1 (191)	28.0 (172)	22.3 (137)	17.6 (108)	<0.001
Hematoma infratentorial % (N)	19.2 (118)	23.4 (144)	22.0 (135)	21.6 (133)	0.338
Hematoma size (mean ± SD)	31.8 ± 28.1	26.1 ± 26.8	23.6 ± 32.9	15.4 ± 19.3	<0.001
GCS (mean ± SD)	8.5 ± 3.9	9.5 ± 4.2	10.7 ± 4.0	12.4 ± 3.6	<0.001
Laboratory tests (mean ± SD)					
Albumin, g/dL	3.6 ± 0.7	3.8 ± 0.7	3.9 ± 0.6	4.0 ± 0.5	<0.001
Ptt	8.7 ± 3.4	8.3 ± 3.5	7.5 ± 3.1	6.5 ± 2.4	<0.001
Aptt	30.2 ± 12.7	28.9 ± 9.1	29.0 ± 9.3	28.2 ± 6.8	0.004
Lymphocyte, 10^9/L	0.5 ± 0.1	0.8 ± 0.1	1.1 ± 0.1	1.7 ± 0.6	<0.001
Monocyte, 10^9/L	0.2 ± 0.1	0.4 ± 0.1	0.6 ± 0.1	1.0 ± 0.3	<0.001
Neutrophil, 10^9/L	5.1 ± 1.1	7.8 ± 0.7	10.4 ± 0.8	15.7 ± 6.8	<0.001
RBC, 10^12/L	3.3 ± 0.4	4.1 ± 0.2	4.5 ± 0.3	5.1 ± 0.5	<0.001
Platelet, 10^9/L	77.4 ± 21.2	125.5 ± 11.8	166.0 ± 12.8	246.0 ± 73.8	<0.001

All continuous variables are presented as mean ± SD, and categorical variables are presented as frequency (%). *p*-values are calculated using ANOVA or Chi-square tests as appropriate. Q1: <5.33; Q2: 5.33–8.95; Q3: 8.95–15.25; Q4: >15.25. ALI, Advanced Lung Cancer Inflammation Index; BMI, body mass index; SBP, systolic blood pressure; GCS, Glasgow Coma Scale; Ptt, prothrombin time; Aptt, activated partial thromboplastin time; RBC, red blood cell count.

**Figure 1:**
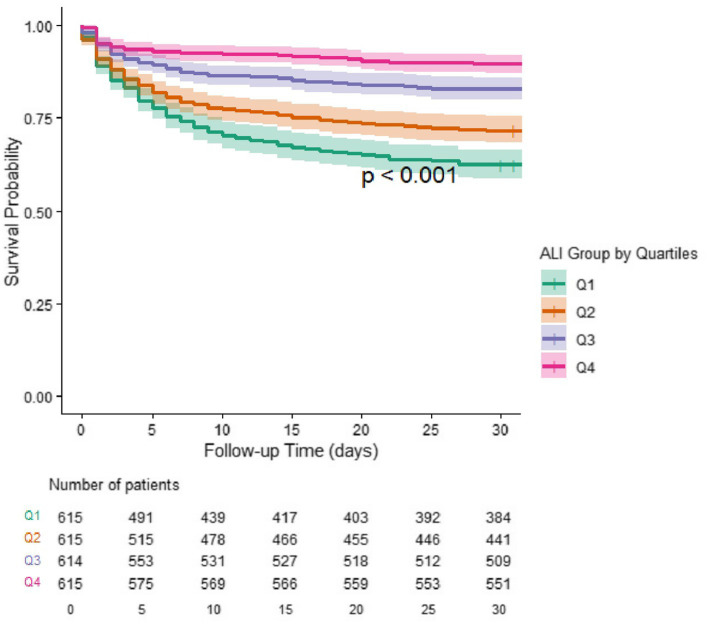
Kaplan–Meier survival curves for 30-day mortality stratified by ALI quartiles. Patients were categorized into four quartiles (Q1–Q4) based on baseline ALI scores. Q1 represents the lowest ALI group and Q4 the highest. A significant difference in survival probabilities was observed across quartiles (log-rank test, *p* < 0.001), with higher ALI quartiles demonstrating improved 30-day survival. Shaded areas represent 95% confidence intervals. The number of patients at risk is shown below the time axis.

The overall 30-day mortality rate was 23.3% (*n* = 574) across the entire cohort. Mortality rates varied significantly across ALI quartiles, with the highest mortality observed in Q1 (37.6%) and the lowest in Q4 (10.4%, *p* < 0.001). Kaplan–Meier survival analysis demonstrated significant differences in 30-day survival probabilities across ALI quartiles (log-rank *p* < 0.001; Figure 1). Patients in Q4 exhibited the highest survival probability at 30 days (89.6%), while Q1 patients experienced the poorest outcomes (62.4%).

Univariate logistic regression analysis identified several variables significantly associated with increased 30-day mortality (*p* < 0.10). These included GCS score, hematoma size, hematoma infratentorial, and hematoma intraventricular. Higher GCS scores were strongly protective, while larger hematoma size, infratentorial hematomas, and hematoma intraventricular were associated with increased mortality risk. Laboratory markers of systemic inflammation, such as higher neutrophil counts and lower albumin levels, were also predictive of worse outcomes (Supplementary Table 1).

In the multivariable logistic regression model, ALI scores remained an independent and significant predictor of 30-day mortality after adjusting for key covariates. Each 1-standard deviation increase in ALI was associated with a 5% reduction in the odds of mortality (*AOR*: 0.95, 95% *CI*: 0.93–0.96, *p* < 0.001). When analyzed as quartiles, patients in Q4 exhibited a 55% reduction in the odds of mortality compared to those in Q1 (*AOR*: 0.45, 95% *CI*: 0.31–0.63, *p* < 0.001; Table 2).

**Table 2:** Multivariable logistic regression analysis for 30-day mortality.

Characteristics	Univariable *OR* (95% *CI*)	*p*-value	Multivariable *AOR* (95% *CI*)	*p*-value
ALI	0.91 (0.89–0.92)	<0.001	0.95 (0.93–0.96)	<0.001
ALI_Low	Ref	–	Ref	–
ALI_High	0.32 (0.27–0.38)	<0.001	0.54 (0.43–0.68)	<0.001
Q1	Ref	–	Ref	–
Q2	0.66 (0.52–0.83)	0.001	0.79 (0.61–1.04)	0.092
Q3	0.34 (0.26–0.45)	<0.001	0.51 (0.38–0.68)	<0.001
Q4	0.19 (0.14–0.26)	<0.001	0.45 (0.31–0.63)	<0.001

Multivariable models were adjusted for age, smoking, alcohol, hypertension, diabetes, GCS score, hematoma size, hematoma infratentorial, and hematoma intraventricular. ALI, Advanced Lung Cancer Inflammation Index; OR, odds ratio; AOR, adjusted odds ratio; CI, confidence interval. ALI_Low (≤ median) and ALI_High (> median). Q1: <5.33; Q2: 5.33–8.95; Q3: 8.95–15.25; Q4: >15.25.

**Figure 2:**
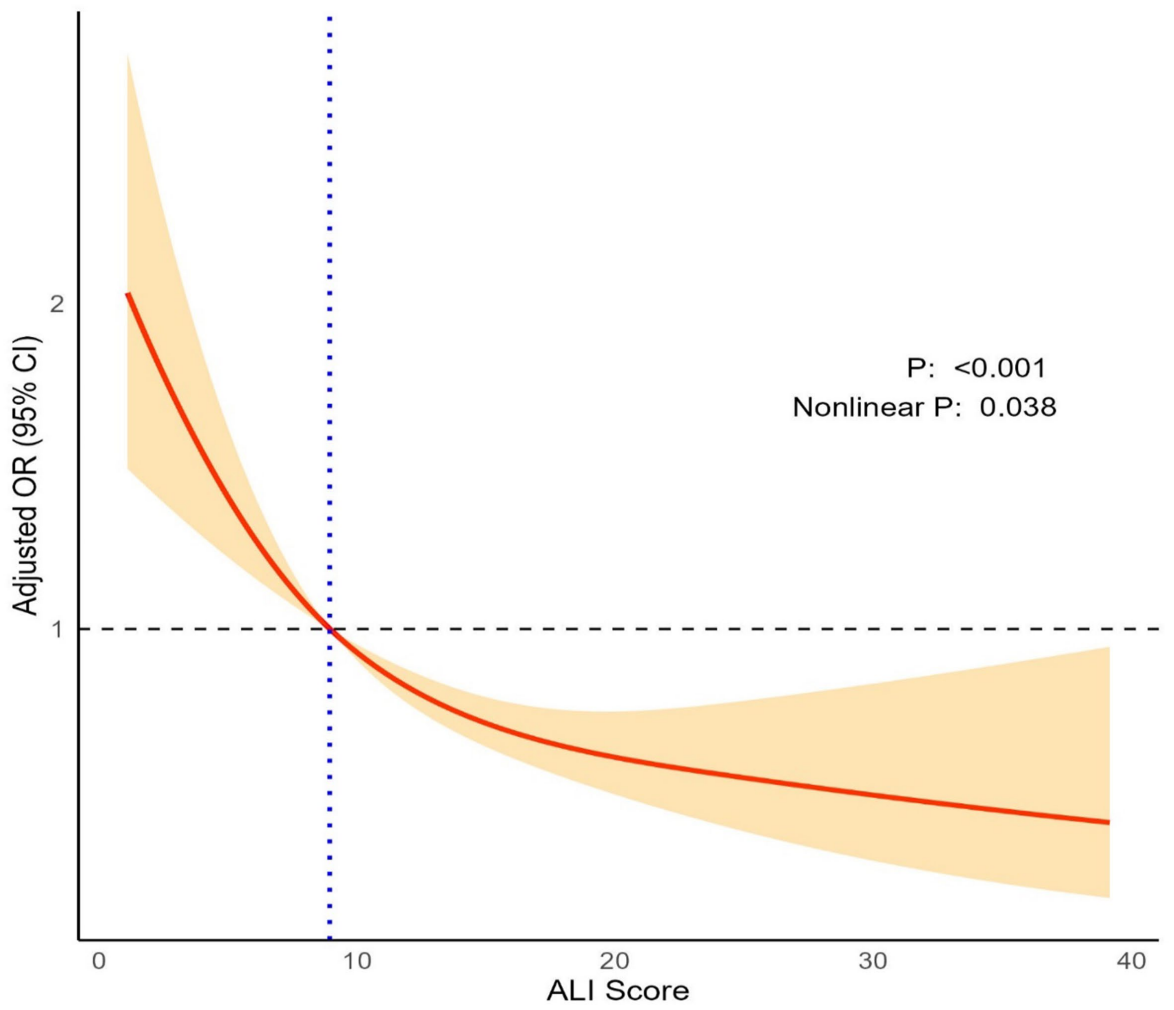
Restricted cubic spline analysis showing the nonlinear association between ALI scores and 30-day mortality. The x axis shows ALI score, and the y axis shows the OR of 30-day mortality. The model was adjusted for age, smoking, alcohol, hypertension, diabetes, GCS score, hematoma size, hematoma infratentorial, and hematoma intraventricular.

Restricted cubic spline analysis demonstrated a nonlinear relationship between ALI scores and 30-day mortality (*p* < 0.001; Nonlinear *p* = 0.038; Figure 2). Adjusted *OR*s decreased significantly as ALI scores increased, with the steepest reduction observed below an ALI score of approximately 8.94, where the *OR* approached 1. Beyond this threshold, the protective effect of higher ALI scores persisted but plateaued at higher scores. This finding highlights a dose-dependent relationship, where increasing ALI is associated with a progressive reduction in 30-day mortality risk.

**Figure 3:**
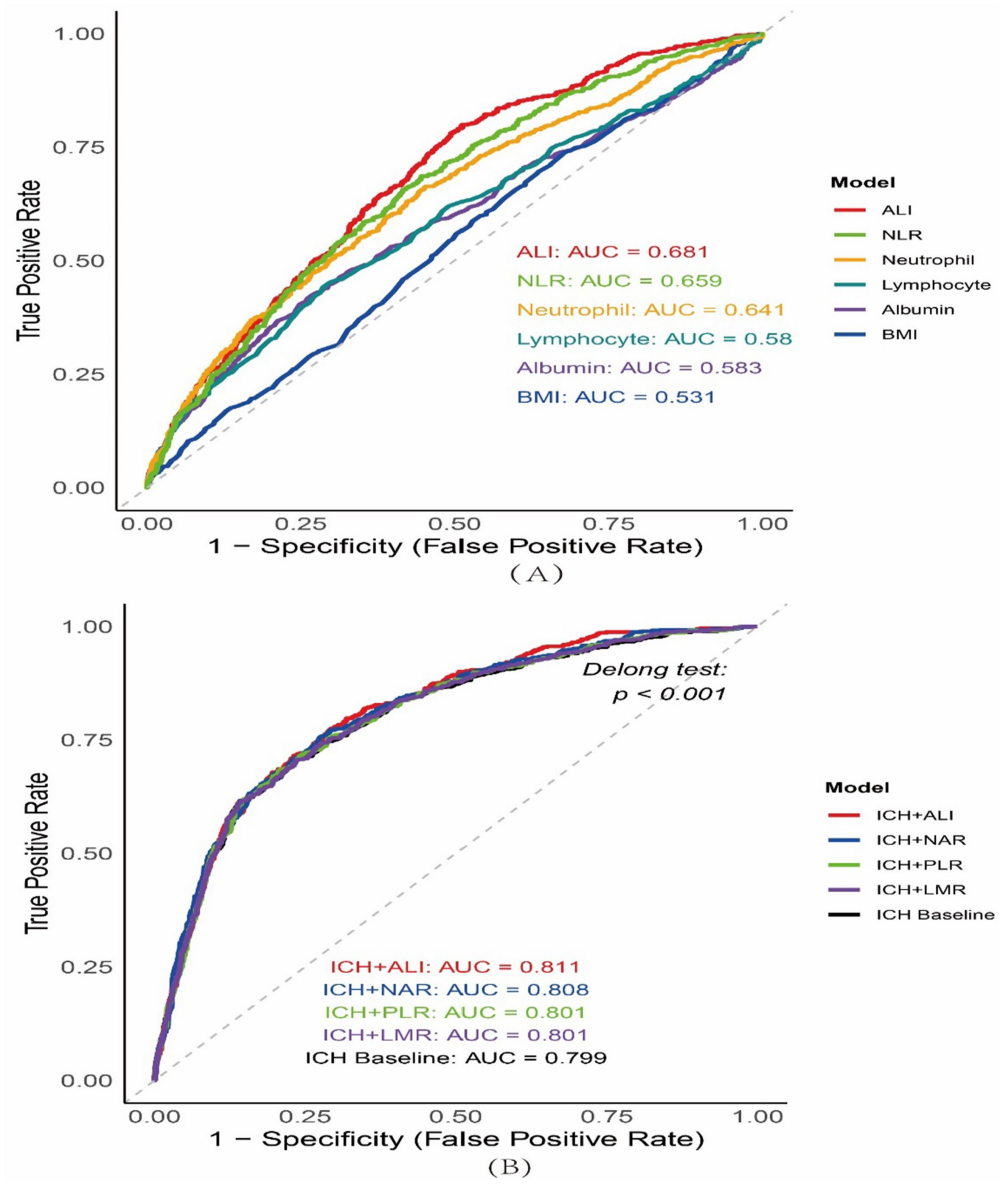
The receiver operating characteristic (ROC) curves. **(A)** ROC curves comparing the discriminatory ability of the Advanced Lung Cancer Inflammation Index (ALI) versus its individual components—body mass index (BMI), serum albumin, neutrophil count, lymphocyte count, and neutrophil-to-lymphocyte ratio (NLR)—for predicting 30-day mortality after intracerebral hemorrhage (ICH). **(B)** ROC curves assessing the added prognostic value of inflammation-based biomarkers (ALI, neutrophil-to-albumin ratio [NAR], platelet-to-lymphocyte ratio [PLR], and lymphocyte-to-monocyte ratio [LMR]) when incorporated into a baseline ICH prediction model. The addition of ALI (red curve, AUC = 0.811) yielded the highest discriminatory performance, followed by NAR (blue curve, AUC = 0.808), PLR (green curve, AUC = 0.801), and LMR (purple curve, AUC = 0.801), compared to the baseline ICH model alone (black curve, AUC = 0.799). Statistical significance of AUC differences was assessed using the DeLong test (*p* < 0.001).

ALI demonstrated superior prognostic value for 30-day mortality. In ROC curve analysis, the standalone ALI score (AUC = 0.681) significantly outperformed its individual components, including BMI (AUC = 0.531), albumin (AUC = 0.583), and NLR (AUC = 0.659) (DeLong test, *p* < 0.001 for all comparisons; Figure 3A).

Importantly, ALI also improved the performance of the established ICH prediction model. Incorporating ALI into the baseline model increased the AUC from 0.799 to 0.811 (*p* < 0.001; Figure 3B), representing a notable enhancement in discrimination. Although other inflammation-based indices showed slight improvements, ALI yielded the greatest gain. To assess the clinical relevance of this improvement, an NRI analysis was conducted. The addition of ALI led to an overall NRI of 3.8% (*p* = 0.018), primarily driven by the correct upward reclassification of 8.4% of non-survivors and downward reclassification of 6.0% of survivors (Supplementary Table 2 and Figure 2).

Baseline characteristics were generally comparable between included and excluded patients, with only modest differences observed in GCS score, hematoma size, and intraventricular hemorrhage, suggesting a low likelihood of selection bias (Supplementary Table 3). Sensitivity analysis using the E-value further supported the robustness of the findings: the E-value was 3.54 (lower limit: 1.98) for ALI quartile 3 and 4.57 (lower limit: 2.25) for quartile 4, indicating that the observed associations are unlikely to be fully explained by unmeasured confounding.

**Figure 4:**
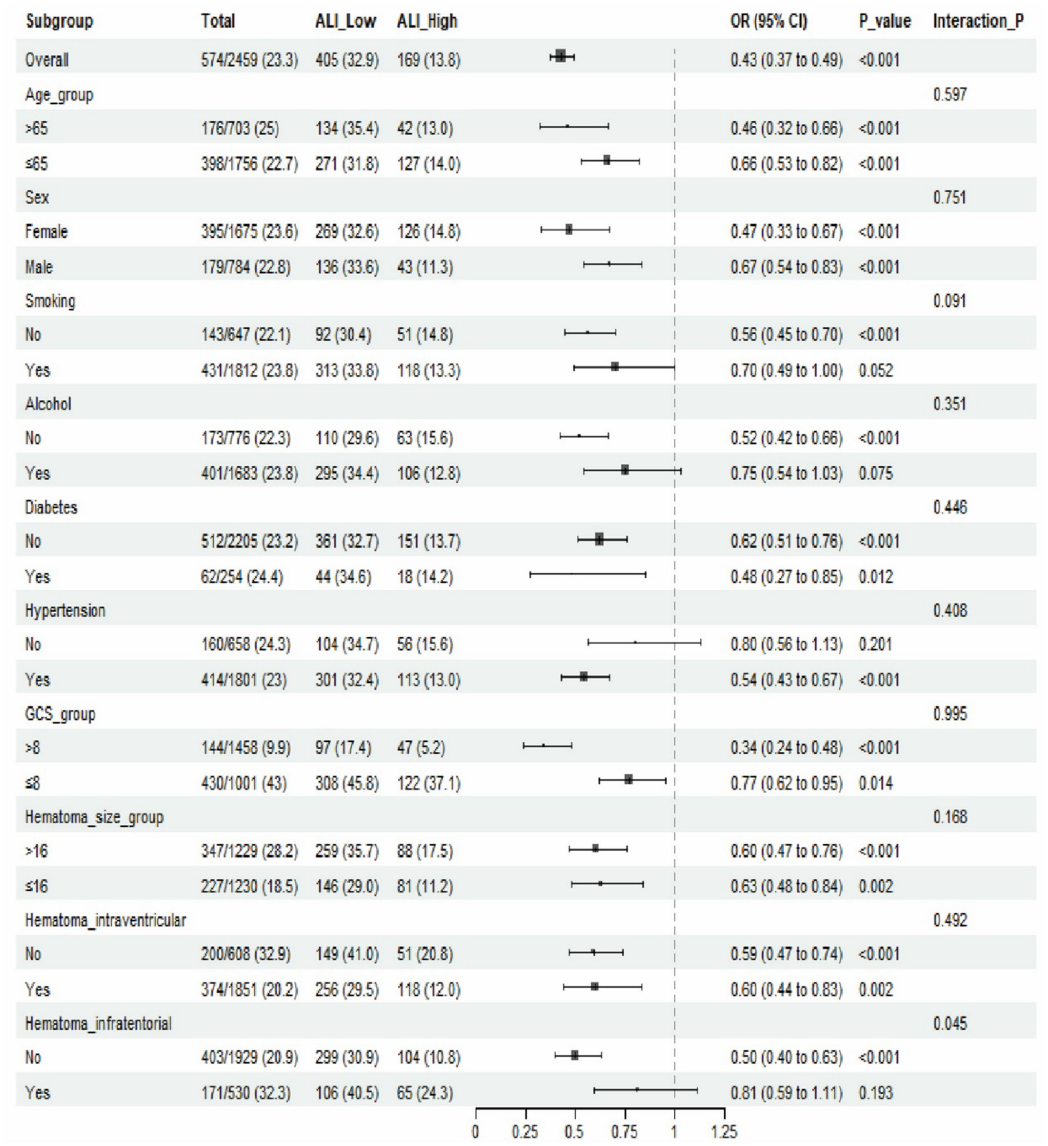
Forest plot summarizing subgroup analysis for the association between ALI and 30-day mortality. Patients were stratified into ALI Low (< median) and ALI High (> median) groups. Subgroup analyses were conducted across clinically relevant variables, including age, sex, comorbidities, hematoma characteristics, and neurological severity. Odds ratios (ORs) with 95% confidence intervals (CIs) were calculated using multivariable logistic regression adjusted for baseline covariates. The protective effect of higher ALI was consistent across all subgroups, with no significant interaction effects (Interaction *p* > 0.05), indicating that the prognostic value of ALI was stable across different clinical strata.

Subgroup analyses confirmed that the association between ALI scores and 30-day mortality was consistent across clinically relevant subgroups (Figure 4). Interaction tests showed no significant modification of the ALI score-mortality relationship by these subgroups (*p* > 0.05).

Notably, the protective effect of higher ALI scores was most pronounced in patients with higher GCS scores and smaller hematoma sizes, suggesting that ALI scores may have the greatest prognostic utility in patients with less severe initial presentations. This consistency across subgroups supports the robustness of ALI scores as an independent predictor of short-term mortality in ICH patients.

## Discussion

This study identifies ALI as a robust and independent prognostic marker for 30-day mortality in ICH patients. Higher ALI scores were significantly associated with reduced mortality risk, with a 55% reduction in the odds of mortality observed in patients within the highest ALI quartile compared to the lowest. Importantly, ALI outperformed its individual components—BMI, serum albumin, and NLR—highlighting its value as a comprehensive biomarker for early risk stratification in neurocritical care. These findings suggest that ALI has the potential to improve clinical decision-making and enhance outcome prediction for ICH patients.

Although originally developed as a prognostic index in oncology, the ALI has gained increasing recognition in neurological diseases due to its ability to integrate systemic inflammation and nutritional reserve—both of which are closely linked to outcomes in neurocritical care. For example, previous research has demonstrated the predictive value of elevated NLR for mortality in ICH, with a higher NLR associated with worse outcomes (8, 24). Similarly, low serum albumin levels have been linked to increased mortality risk, primarily due to their role in maintaining oncotic pressure and modulating inflammation (25). BMI, while associated with outcomes in certain populations, has shown inconsistent prognostic value, particularly in the context of critical illness (26). Recent studies have shown that lower ALI scores are independently associated with increased infarct volumes, poor functional recovery, and higher short- and long-term mortality in ischemic stroke and ICH cohorts (14, 27, 28). These findings underscore the broader applicability of ALI beyond its oncologic origins.

Collectively, these findings emphasize the intertwined roles of systemic inflammation and malnutrition in driving ICH pathophysiology. ALI appears particularly valuable as a composite biomarker that captures the synergistic impact of these two interrelated pathophysiological processes. Unlike individual markers such as NLR or serum albumin, ALI provides a more comprehensive reflection of both systemic inflammatory burden and nutritional reserve, both of which contribute significantly to the development of secondary brain injury.

Mechanistically, systemic inflammation and malnutrition are considered parallel biological stressors that jointly exacerbate neuronal injury following ICH (5). Inflammatory cytokines such as TNF-*α*, IL-1β, and IL-6, released by activated immune cells, compromise the integrity of the blood–brain barrier, promote cerebral edema, and trigger neuronal apoptosis (29, 30). In parallel, nutritional deficiency impairs mitochondrial function, weakens immune defense, and reduces the capacity for tissue repair (31). Previous studies have linked poor nutritional status to increased hematoma expansion and mortality, underscoring its clinical relevance. By capturing both inflammatory and nutritional imbalances, ALI may reflect the overall systemic vulnerability of patients with ICH. This interpretation is consistent with growing evidence in other critical illnesses such as sepsis and ischemic stroke, where the interaction between inflammation and nutrition plays a key role in prognosis (14, 32, 33).

The findings support the rationale for adopting ALI in neurocritical care: by combining nutritional reserve and systemic inflammation, ALI provides a composite snapshot of the host’s physiological reserve. Our study reinforces this approach by showing that ALI independently predicts 30-day mortality in ICH patients, extending its prognostic utility beyond its original oncologic context.

Furthermore, our study aligns with recent advances in the use of composite biomarkers to improve risk stratification in neurocritical care. A recent meta-analysis highlighted the importance of combining inflammatory and nutritional markers to capture the multifaceted nature of critical illness, echoing the rationale behind ALI (34). Unlike these earlier studies, our research specifically applies ALI to ICH patients, providing new insights into its role in this population.

The potential clinical utility of ALI lies in its dual strengths: simplicity of calculation from routine laboratory data and its demonstrated ability to refine risk prediction. Although the absolute improvement in AUC after adding ALI to the baseline ICH score was modest (from 0.799 to 0.811), this metric alone may underestimate its clinical value. Our NRI analysis provides more granular insight. A statistically significant NRI of 3.8% confirms that ALI correctly reclassifies a meaningful proportion of patients into more accurate risk strata. In a high-stakes environment like neurocritical care, even such enhancements in early risk stratification can be impactful—potentially informing decisions on ICU monitoring intensity, guiding family counseling on prognosis, or identifying eligible candidates for clinical trials. This highlights the importance of using reclassification metrics to complement traditional discrimination statistics when evaluating novel biomarkers.

However, translating ALI into a bedside clinical tool requires the establishment of a clear, actionable threshold. Our restricted cubic spline analysis revealed a nonlinear relationship with an inflection point near 8.94, below which mortality risk rises sharply. While statistically informative, this data-driven point cannot be directly adopted as a universal cutoff without rigorous validation. Therefore, our use of quartile-based stratification, a common approach in exploratory biomarker research (14), serves to demonstrate a dose–response relationship but does not define a clinical decision point. A critical next step is to conduct large-scale, prospective studies aimed specifically at defining and validating optimal ALI cutoffs. Such studies are essential to unlock the full translational potential of ALI, allowing it to be readily applied to guide personalized management in diverse ICH populations.

This study has several strengths. The large sample size and comprehensive data spanning a decade enhance statistical power and generalizability, while rigorous statistical methods, including logistic regression and restricted cubic spline analyses, provide a robust and nuanced evaluation of ALI’s prognostic value. Furthermore, the integration of ALI into an established ICH prediction model highlights its translational potential for clinical application.

Despite these strengths, this study has several limitations that must be acknowledged. First, as a single-center retrospective study, it is inherently susceptible to selection bias and residual confounding. Although selection bias due to missing data cannot be completely excluded, baseline comparability suggests its potential influence. Second, the decade-long study period (2009–2019) introduces the possibility of time-related confounding due to advancements in neurocritical care. We believe this risk is mitigated because ALI is calculated from baseline variables reflecting the patient’s pre-intervention state, and core management protocols at our institution remained stable. Nonetheless, subtle shifts in practice cannot be entirely ruled out, underscoring the need for validation in contemporary cohorts. Third, our primary outcome was 30-day mortality. While a critical endpoint, this does not capture other important long-term outcomes, such as functional recovery, cognitive status, or quality of life, which are of great importance to patients and families. Finally, practical considerations may affect the widespread implementation of ALI. Its calculation relies on timely and accurate measurements of BMI, albumin, and NLR, which may be challenging in acute emergency settings or in resource-constrained healthcare systems. Given these limitations, large-scale, prospective, multicenter studies are imperative. Such research is needed not only to validate our findings and confirm the generalizability of ALI but also to assess its impact on long-term outcomes and its utility in guiding targeted interventions in real-world clinical practice.

## Conclusion

Our findings indicate that a higher ALI is significantly associated with reduced 30-day mortality in patients with ICH, establishing ALI as an independent and robust prognostic marker. By integrating inflammatory and nutritional parameters, ALI enables early risk stratification and supports targeted interventions. As a simple and accessible index, ALI holds promise for improving patient management and outcomes. Further studies are required to validate its clinical utility.

## Data Availability

The raw data supporting the conclusions of this article will be made available by the authors, without undue reservation.

## Footnotes

^1^ https://www.evalue-calculator.com/evalue
