# Supplementary material for: Association between Advanced Lung Cancer Inflammation Index and 30-day mortality in patients with spontaneous intracerebral hemorrhage: a retrospective cohort study
Source: Front Neurol. 2025 Jul 28;16:1612333. doi: 10.3389/fneur.2025.1612333 (PMC12336023; doi:10.3389/fneur.2025.1612333)
Supplement: Supplementary file 1 [file Image_1.pdf]

Supplementary Table 1

Characteristics	Univariable *OR* (95% *CI*)	*P*-value	Multivariable *AOR* (95% *CI*)	*P*-value
ALI	0.91 (0.89–0.92)	<0.001	0.95(0.93–0.96)	<0.001
ALI_Low	Ref	-	Ref	-
ALI_High	0.32 (0.27–0.38)	<0.001	0.54 (0.43–0.68)	<0.001
Q1	Ref	-	Ref	-
Q2	0.66 (0.52–0.83)	0.001	0.79 (0.61–1.04)	0.092
Q3	0.34 (0.26–0.45)	<0.001	0.51 (0.38–0.68)	<0.001
Q4	0.19 (0.14–0.26)	<0.001	0.45 (0.31–0.63)	<0.001
Age	1.00 (0.99–1.01)	0.276	1.01（1.00-1.01）	0.073
Sex	1.04 (0.94–1.15)	0.646	NA	NA
Smoking	0.92 (0.82–1.03)	0.352	0.90（0.67-1.20）	0.411
Alcohol	0.92 (0.83–1.02)	0.349	0.96（0.73-1.26）	0.764
BMI	0.97 (0.96–1.01)	0.009	0.99 (0.97–1.02)	0.095
SBP	1.00 (0.99–1.00)	0.001	1.00 (0.99–1.00)	<0.001
Diabetes	1.07 (0.92–1.23)	0.600	0.94（0.66-1.34）	0.734
Hypertension	0.91 (0.80–1.03)	0.301	0.79（0.62-1.01）	0.064
Hematoma	1.94 (1.58–2.37)	<0.001	1.39 (1.10–1.77)	0.006
Hematoma	1.80 (1.46–2.23)	<0.001	1.60 (1.23–2.07)	<0.001
Hematoma size	1.01 (1.01–1.01)	<0.001	1.01 (1.00–1.01)	<0.001
GCS	0.76 (0.74–0.78)	<0.001	0.82 (0.79–0.84)	<0.001
Ptt	1.22 (1.18–1.26)	<0.001	1.16 (1.12–1.21)	<0.001
Aptt	1.04 (1.03–1.06)	<0.001	1.03 (1.01–1.04)	<0.001
Monocyte	1.62 (1.24–2.12)	<0.001	1.34 (0.98–1.82)	0.065
Lymphocyte	0.73 （0.61-0.87）	<0.001	-	-
Platelet	0.99（0.99-1.00）	<0.001	1.00 (1.00, 1.00)	0.006
Neutrophil	1.13 (1.10–1.15)	<0.001	-	-
Albumin	0.60 (0.52–0.70)	<0.001	-	-

Q1:< 5.33; Q2:5.33-8.95; Q3:8.95-15.25; Q4:>15.25 ALI, Advanced Lung Cancer Inflammation Index; BMI, Body Mass Index; SBP, Systolic Blood Pressure; GCS, Glasgow Coma Scale; Ptt, Prothrombin Time; Aptt, Activated Partial Thromboplastin Time; RBC, Red Blood Cell Count.

Supplementary Table 2

**Characteristics**	**Estimate**	**95% *CI***	***P*-value**
Among patients who died (Cases, n=574)			
Correct upward reclassification (%)	8.4%		
Incorrect downward reclassification (%)	4.2%		
Net Improvement (NRI+)	4.2%		
Among patients who survived (Controls, n=1885)			
Correct downward reclassification (%)	6%		
Incorrect upward reclassification (%)	6.4%		
Net Improvement (NRI-)	-0.4%		
Overall Net Reclassification Improvement (NRI)	3.8%	0.7 -7.2	0.018

Data are presented as percentages (%). The analysis compares a baseline model (including age, GCS, hematoma size, intraventricular hemorrhage, and infratentorial location) with a new model that adds the Advanced Lung Cancer Inflammation Index (ALI). Reclassification was based on three predefined risk categories for 30-day mortality: Low (<10%), Intermediate (10-40%), and High (≥40%). Detailed patient counts for the reclassification are visualized in Supplementary Table 2. NRI+ and NRI- represent the Net Reclassification Improvement for cases (deaths) and controls (survivors), respectively. A positive value indicates improved classification. CI, confidence interval.

Supplementary Table 3

Characteristics	Included Group (N = 2459)	Excluded Group (N = 3795)	*P*-value
Age, years (mean ± SD)	56.5 ± 14.0	57.0 ± 14.4	0.152
GCS (mean ± SD)	11.4 ± 3.1	10.3 ± 3.4	<0.001
Hematoma size, mL (mean ± SD)	21.4 ± 17.5	24.2 ± 20.3	<0.001
Hypertension, n (%)	1330 (54.1%)	1885 (49.7%)	0.001
Diabetes, n (%)	412 (16.8%)	637 (16.8%)	0.461
Hemorrhage intraventricular, n (%)	823 (33.5%)	1438 (37.9%)	<0.001
Hemorrhage infratentorial, n (%)	250 (10.2%)	404 (10.6%)	0.459

Comparison of baseline characteristics between included and excluded patients.

This table presents a comparison of key clinical and demographic variables between patients included in the final analysis (n = 2,459) and those excluded due to missing data (n = 3,795). Variables assessed include age, Glasgow Coma Scale (GCS) score, hematoma size, and the presence of comorbidities and hematoma location.

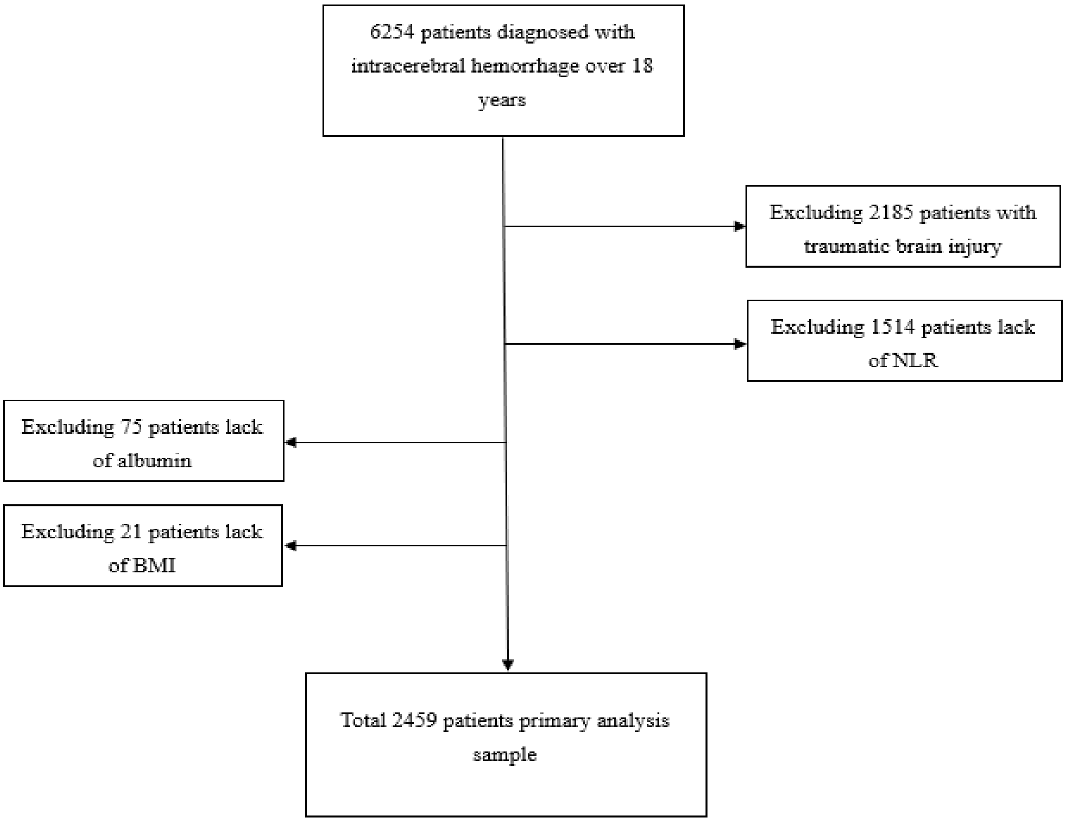

Supplementary Figure 1 Study flow chart showing the inclusion and exclusion criteria.

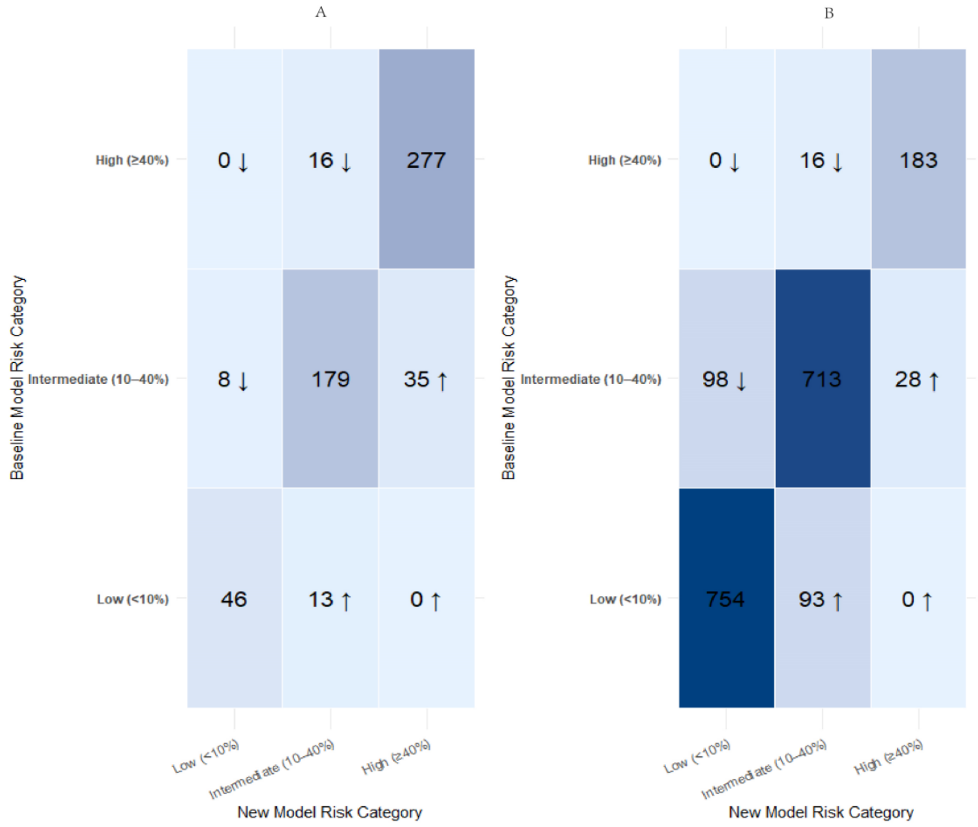

Supplementary Figure 2 Reclassification matrix for 30-day mortality by adding ALI to the baseline model.

(A) Reclassification matrix for patients who died (n = 574).(B) Reclassification matrix for patients who survived (n = 1,885).Cells show the number of patients reclassified across risk categories between the baseline model and the new model. Arrows indicate the direction of reclassification (↑ upward, ↓ downward). Shading intensity corresponds to patient count. Risk categories are defined as Low (<10%), Intermediate (10–40%), and High (≥40%) predicted risk of the outcome.
